# Evolution of Gene Expression Balance Among Homeologs of Natural Polyploids

**DOI:** 10.1534/g3.116.038711

**Published:** 2017-02-13

**Authors:** Jasdeep S. Mutti, Ramanjot K. Bhullar, Kulvinder S. Gill

**Affiliations:** Department of Crop and Soil Sciences, Washington State University, Pullman, Washington 99164-6420

**Keywords:** dosage dependence, evolution of new gene functions, homoeologous gene expression, polyploidy, wheat

## Abstract

Polyploidy is a major evolutionary process in eukaryotes, yet the expression balance of homeologs in natural polyploids is largely unknown. To study this expression balance, the expression patterns of 2180 structurally well-characterized genes of wheat were studied, of which 813 had the expected three copies and 375 had less than three. Copy numbers of the remaining 992 ranged from 4 to 14, including homeologs, orthologs, and paralogs. Of the genes with three structural copies corresponding to homeologs, 55% expressed from all three, 38% from two, and the remaining 7% expressed from only one of the three copies. Homeologs of 76–87% of the genes showed differential expression patterns in different tissues, thus have evolved different gene expression controls, possibly resulting in novel functions. Homeologs of 55% of the genes showed tissue-specific expression, with the largest percentage (14%) in the anthers and the smallest (7%) in the pistils. The highest number (1.72/3) of homeologs/gene expression was in the roots and the lowest (1.03/3) in the anthers. As the expression of homeologs changed with changes in structural copy number, about 30% of the genes showed dosage dependence. Chromosomal location also impacted expression pattern as a significantly higher proportion of genes in the proximal regions showed expression from all three copies compared to that present in the distal regions.

Polyploidy, or whole genome duplication, has been the primary force shaping the evolution and success of flowering plants probably by providing genome buffering against deleterious mutations, increasing allelic diversity, increasing heterozygosity (heterosis), and by evolving novel phenotypes (Stebbins 1950; Grant 1981; Comai 2005). Inferred either by comparison of chromosome numbers (Stebbins 1950) or by bio-systematic approaches (Masterson 1994; Grusz *et al.* 2009; Buggs *et al.* 2011), 57–70% of flowering plants are reported to be polyploids. Currently available sequencing data have elucidated a much broader role of polyploidy in evolution (Doležel *et al.* 2012; Kellogg 2015; Ming and Wai 2015). Even the well-known diploids including *Arabidopsis*, humans, maize, rice, soybean, tomato (Blanc and Wolfe 2004), and unicellular organisms such as yeast and the ciliate *Paramecium tetraurelia*, are also believed to have undergone cycles of ancient or contemporary polyploidization (Wolfe and Shields 1997; Venter *et al.* 2001; Aury *et al.* 2006).

Based on the origin and level of ploidy, the polyploids can be classified as an auto- (doubling of a diploid genome) or allopolyploid (chromosome doubling of an interspecific hybrid). Doubling of genetic material in a nucleus of either the same or related genomes comes as a major genomic stress that has to be alleviated by structural and/or gene expression changes. Duplicated genes in a recent polyploid can have several different fates, including the silencing of one of the duplicated copies (nonfunctionalization), divergence leading to new functions (neofunctionalization), maintenance of expression at the diploid level, or the acquisition of different tissue specificities (subfunctionalization) (Lynch and Conery 2000; Roulin *et al.* 2013; Li *et al.* 2015). Among these, subfunctionalization and nonfunctionalization are the predominant fates as shown in *Arabidopsis* (Jeffrey Chen *et al.* 2004; Groszmann *et al.* 2011), *Tragopogon mirus* (Buggs *et al.* 2010), *P. tetraurelia* (Arnaiz *et al.* 2010), *Xenopus laevis* (Sémon and Wolfe 2008), human (Gu *et al.* 2002), soybean (Roulin *et al.* 2013), and cotton (Adams *et al.* 2003; Adams and Wendel 2005; Chaudhary *et al.* 2009). Structural changes at both the chromosome and gene level have also been reported. These include homeolog-specific gene loss in the ancestral tetraploid *Arabidopsis* (Thomas *et al.* 2006) and recently formed natural allotetraploid *T. mirus* (Koh *et al.* 2010); ancient and ongoing targeted gene loss in maize (Schnable *et al.* 2011); gene elimination in wheat (Feldman *et al.* 1997; Shaked *et al.* 2001; Mochida *et al.* 2003) and *Tragopogon* (Tate *et al.* 2006); and chromosomal translocation and transposition in *Brassica* allopolyploids (Song *et al.* 1995). Recent advances in transcriptome profiling have confirmed these conclusions (Bombarely *et al.* 2014). However, the fate of duplicated genes over time and in different tissues and developmental stages of natural polyploids, such as wheat, has not been extensively studied, although expression level change in resynthesized allohexaploid wheat (Akhunova *et al.* 2010) along with partial to complete genome dominance has been reported (Pfeifer *et al.* 2014).

Several different molecular processes have been reported to be responsible for various fates of duplicated genes, some of which are: (i) altered methylation pattern, as was observed for 30% of the allopolyploid *Spartina* genes (Salmon *et al.* 2005); (ii) heterochromatin disruption and altered imprinting as observed in *Arabidopsis thaliana* × *A. arenosa* allopolyploids; and (iii) activation of transposons as observed in the synthetic allotetraploids of both *Arabidopsis* and wheat (Kashkush *et al.* 2003; Comai 2005; Salmon *et al.* 2005). Many of these studies were conducted on synthetic polyploids, thus, the reported effects may be the immediate responses of the plant to the stress caused by wide hybridization or doubling of the genetic material rather than the largely unknown effects of polyploidization over time. These effects were reported in some of the paleopolyploids but are not well-known in intact polyploids like wheat.

Differences in both the level of expression as well as the tissue specificity of duplicated genes are well-documented. Expression patterns of 199 of the 461 (43%) genes of natural allopolyploid cotton were different among homeologs (Udall and Wendel 2006). Expression pattern differences and differences in tissue specificities were confirmed in the follow-up studies (Yoo *et al.* 2013; Yoo and Wendel 2014). Similarly, RNA-seq expression analysis of ∼18,000 duplicated genes in Soybean (*Glycine max* L.) showed differential expression patterns in ∼50% of the paralogs (Roulin *et al.* 2013). However, it is difficult to determine if the observed expression differences were the result of polyploidization or differences among independently evolving species with different evolutionary distances and expression patterns. The polyploidization in cotton is thought to have occurred ∼1–2 million years ago, while the soybean genome underwent two separate polyploidization events 13 and 59 million years ago (Wendel and Cronn 2003; Roulin *et al.* 2013).

Similar analyses in relatively “younger” polyploids such as wheat are poorly studied. The *TaBx* gene, for example, displays a tissue-specific differential expression pattern, with the B genome copy expressed more in shoots as compared to the other two homeologs (Nomura *et al.* 2005). One homeolog each was shown to be silenced in 236 unigenes, expressing in leaf and root tissues of wheat (Bottley *et al.* 2006). Tissue-specific homeologous silencing was also shown in 13 homeologous pairs of allotetraploid *T. mirus* (Buggs *et al.* 2010). However, no generalization can be made based on data from a single gene or subset of unigenes expressing in two wheat tissues.

Various chromosomes and chromosomal regions are structurally and functionally distinct, as evident from the fact that both the genes as well as recombination are highly uneven along the eukaryotic chromosomes (Gill *et al.* 1993; Sidhu and Gill 2005; Schnable *et al.* 2011). The relationship between this uneven distribution of genes and recombination with the differential fate of duplicated genes has not yet been established. In the present study, we have shown that the location of a duplicated gene on a chromosome/arm/region impacts the evolution of its gene expression pattern.

Here, we chose wheat to study the fate of duplicated genes in natural polyploids. Wheat, *Triticum aestivum* L. (2*n* = 6*x* = 42), is an allohexaploid with three relatively collinear genomes, designated A, B, and D (Kihara 1944; McFadden and Sears 1946; Feldman *et al.* 1966). Two independent polyploidization events led to the evolution of hexaploid wheat. The first polyploidization step occurred ∼500,000-yr-ago from a hybridization event between *Tr. urartu* Tumanian ex Gandilyan (2*n* = 14, genome AA) and *Aegilops speltoides* Tausch (2*n* = 2*x* = 14, genome SS≈BB) or its closely related species, giving rise to the allotetraploid *Tr. turgidum* L. (McFadden and Sears 1946; Huang *et al.* 2002). The second polyploidization step happened ∼8000-yr-ago from a hybridization event between *T. turgidum* L. (2*n* = 28, AABB) and *Ae. tauschii (Coss.) Schmal* (*2n* = 14, DD), giving rise to the present day hexaploid wheat (Kihara 1944). Furthermore, the diploid progenitors of hexaploid wheat are extant and their phylogenetic relationship is relatively well-understood (Sasanuma *et al.* 1996; Huang *et al.* 2002). Also available in hexaploidy, wheat is a wealth of aneuploid stocks (Sears 1954; Sears and Sears 1978; Endo 1988; Endo and Gill 1996) that are ideal to understand the functional organization of the wheat genome and to study the effect of polyploidy on gene expression over time. Therefore, the objectives of the present study were to study fate of duplicated genes in wheat, establish the relationship of relative chromosomal location with the evolution of gene expression balance, the differential evolution of tissue specificity among duplicated genes, and the extent of dosage effect on gene expression.

## Materials and Methods

### Plant material

Wheat nullisomic–tetrasomic lines (NT, these lack a pair of chromosomes, the deficiency of which is compensated for by a double dose of either of the two homeologous chromosomes) and ditelosomic lines (DT, lack a pair of chromosome arms) produced in cultivar “Chinese spring” (CS) (Sears 1954), along with cultivar CS, grown at greenhouse conditions, were used for various experiments. For methylation studies and single-stranded conformational polymorphism (SSCP) analysis, plants were grown under highly controlled conditions in a growth chamber [18/6 hr (day/night) light and 22/18° (day/night) temperature]. Plants were started on the same day, spaced equally, and were provided with equal amounts of water at regular intervals. The plant material was obtained from the Wheat Genetics Resource Center (Kansas State University).

### DNA and RNA procedures

The DNA extractions and gel blot analysis procedures were carried out as previously described (Sandhu *et al.* 2001; Erayman *et al.* 2004). Total RNA was extracted using the guanidinium thiocyanate-cesium chloride density gradient method (Dilbirligi *et al.* 2004; Mutti *et al.* 2010). The poly(A)^+^ RNA was selected from total RNA by affinity chromatography on oligo(dT) cellulose using the standard protocol. The hot phenol RNA extraction method was used when only a small quantity of RNA was needed for SSCP analysis. For homeologous gene expression analysis using SSCP analysis, RNA from 11 different plant development stages [namely 5-d-old seedling, root from seedling and adult plants, 28-d-old plant, flag leaf (last leaf that is visible but still rolled up), early flowering (Feekes scale: 6, first node of stem visible at base of shoot) (Large 1954), meiosis, preanthesis, postanthesis, seed at 5 days postanthesis (DPA) and 30 DPA stage, and adult plant (Feekes scale: 10.5, all heads out of sheath)] was extracted. The Feekes scale is a system to identify the growth and development of cereal crops. The different plant development stages are represented on a scale from 1 to 11.

### DNA methylation analysis

Gel blot analysis was used to evaluate changes in overall gene expression due to changes in the homeolog copy number. Genomic DNA was extracted from 28-d-old leaf tissue of CS and wheat homeologous group 7 NT and DT lines (N7AT7B, N7AT7D, N7BT7A, N7BT7D, N7DT7A, N7DT7B, DT7AL, DT7AS, DT7BL, DT7BS, and DT7DS). The genomic DNA was cut with restriction enzymes sensitive to CpG and CpNpG methylation (*Hpa*II) and insensitive to internal methylation (*Msp*I). Gel blot analysis was performed using 15 μg of genomic DNA and size separated on 0.8% agarose gels. All other steps were followed as previously described (Gill and Gill 1994).

Methylation studies were conducted using 66 randomly selected wheat homeologous chromosome 7-specific DNA probes. An effort was made to select probes uniformly distributed over the group 7 chromosome. These DNA probes included 50 ESTs present on various wheat genetic maps (http://wheat.pw.usda.gov/GG2/index.shtml). All of the probes used for the study were ESTs except for 16 group 7 probes that were previously identified by RNA fingerprinting/differential display or cDNA-AFLP display of CS and the 7AL-9 (FL0.94) deletion line (Sidhu and Gill 2005). As a control to see whether change in gene dosage of one chromosome has any effect on the genes on other chromosomes of wheat, 12 randomly selected group 5 probes (*abg314*, *abg387*, *wg530*, *mwg522*, *mwg52*, *mwg592*, *mwg2237*, *ksuG414*, *mwg768*, *mwg923*, *bcd508*, and *cdo1090*) and five group 1 probes (*BE590674*, *BE495292*, *BE443071*, *BE443409*, and *BE490291*) were also used.

### SSCP analysis

SSCP analysis was used to resolve fragment bands corresponding to the three homeologs. We optimized the procedure of (Cronn and Adams 2003) for use in wheat. Briefly, first-stranded cDNA was synthesized from 2 μg of DNaseI-treated pooled poly(A)^+^ RNA using Moloney Murine Leukemia Virus reverse transcriptase (Clontech Lab). The PCR reactions were performed with Advantage PCR Kits and Polymerase Mixes (Clontech, Catalog #639101), in the presence of S^35^dATP in a total volume of 20 μl. The PCR product was mixed with an equal volume of loading buffer. About 5 μl of this mixture was loaded onto 0.4 mm thick denaturing 8% polyacrylamide/8 M urea gels. Gels contained and were run in 0.5 × TBE buffer at pH 8.3. Each sample was size separated both on gels run under standard conditions and on gels run for SSCP. For standard runs, the gels were prerun at a 33 mA constant current for 30 min and then at 70 W constant power for 4 hr. For SSCP runs, the gels were prechilled at 4° for at least 5–6 hr before running at 10 W for 12–13 hr at 4°. An X-ray film was placed on the dried gels and was exposed for 3–7 d.

### Identification of wheat-specific EST contigs

Unique ESTs corresponding to 9400 wheat unigenes have been physically mapped to wheat chromosomes by gel-blot DNA analysis of 164 wheat aneuploid lines including NT, DT, and deletion lines (http://wheat.pw.usda.gov/NSF/). Each of the 9400 gel-blot analysis images was manually evaluated to select 2180 that showed a clear hybridization pattern and where every restriction fragment band was physically localized to a chromosomal region. Rice orthologs for the selected wheat ESTs were identified by pairwise comparison of the EST sequences with that of the full-length rice cDNAs using the “Blast” algorithm performed at a cut off e-value of e^−70^ (KOME, http://cdna01.dna.affrc.go.jp/cDNA/). Sequences of the selected wheat ESTs and the corresponding rice full-length cDNAs were then compared with the CS wheat-specific EST database (by limiting query to “wheat and Chinese spring” using the “megablast” algorithm http://www.ncbi.nlm.nih.gov/BLAST/). The identified CS wheat-specific EST sequences aligned with the corresponding wheat and rice sequences were assembled into contigs using the “Contig Express” Module of the Vector NTI Advance10.0 software with ≥ 80% identity and a minimum overlap of 30 bp. These two comparisons yielded CS wheat-specific EST contigs for each of the selected genes.

### Virtual northerns for tissue-specific expression analysis

This method is a relatively easier and more versatile tool of potentially universal application. However, the quality of the virtual northern blots depends on the quality of the EST database used. Differential expression of homeologs in different tissues was studied by manually performing virtual northern blots (Fielden *et al.* 2002; Keller *et al.* 2006) on 220 genes with three structural copies (homeologous), one each for the three genomes. All wheat ESTs (268,582) of the same and different accessions that were developed from 41 total cDNA libraries from 14 different tissues (http://www.ncbi.nlm.nih.gov/dbEST/) were used for this analysis. The three homeologs of a gene were identified by sequence comparison of EST sequences for each gene, and the source tissue for each of the ESTs representing different homeologs was scored. The virtual northern results were confirmed by SSCP analysis of a few randomly selected genes.

### Heat map for tissue-specific expression analysis

Stand-alone batch blast was performed using the FASTA sequences of wheat ESTs that were part of the virtual northern blot analysis, with the reference International Wheat Genome Sequencing Consortium (IWGSC) gene models from Ensembl Plants containing 103,274 genes, at an *e*-value of ≤ 1e−100 to retrieve all the gene copies each having an Ensembl ID (TRAES) number. Only the copies with ≥ 95% identity were retained for further analysis. The expression profiles of all the copies of these genes in three stages, each with three replicates, of each of the five different developmental tissues were analyzed using the available RNA-seq data (Choulet *et al.* 2014) contained in the wheat expression database (http://wheat.pw.usda.gov/WheatExp/). The differential expression analysis was performed utilizing the mean FPKM values of each tissue generated from the wheat expression database and plotted using the ggplot2 package of R software (ver. 3.0.1) with the criterion of fold change.

### Expressed homeologs distribution

Consensus physical maps for seven groups of six chromosomes (one chromosome pair being derived from each of the three ancestral genomes) of wheat were downloaded (Conley 2004; Hossain 2004; Miftahudin *et al.* 2004; Munkvold 2004; Peng 2004; Randhawa 2004). The physical size of the chromosome and chromosome arm were taken, and the physical size of the chromosome intervals (bins) was calculated on the basis of their relative fraction length (FL) (Endo and Gill 1996). The distribution of expressed homeologs was assumed to be uniform along the physical length of a chromosome and chromosome arm. The mean number of expressed EST loci per micrometer of chromosome arm was calculated by dividing the total EST loci mapped to the chromosome arm by the total physical length of that arm (Endo and Gill 1996). The numbers of expected expressed homeologs (all three expressed, two of three expressed, and only one of three expressed) per chromosome bin were calculated by multiplying the mean number of the expressed ESTs with its physical length. The ratio of observed *vs.* expected EST loci was used to estimate the distribution of expressed homeologs. The χ^2^ goodness of fit test was used to detect significant differences between observed and expected numbers of expressed EST loci. In χ^2^ distribution analysis, *P* < 0.05 was considered significant.

### Data availability

The data necessary for supporting the conclusions presented in the article are represented within Supplemental Material, File S1. Figure S1 in File S1 shows expression pattern summary of genes with three structural copies along with the chromosomes. Figure S2 in File S1 shows the genes (with IDs) that will be useful for the readers to follow-up on any of the genes. It shows a consensus expression map of 1030 ESTs with three structural copies physically localized to chromosome regions bracketed by wheat deletion breakpoints. Figure S3 in File S1 shows the methylation pattern between CS and aneuploid stocks of homeologous group 7 chromosomes using methylation-sensitive and -insensitive enzymes.

## Results

### Expression pattern of the homeologs

**Figure 1:**
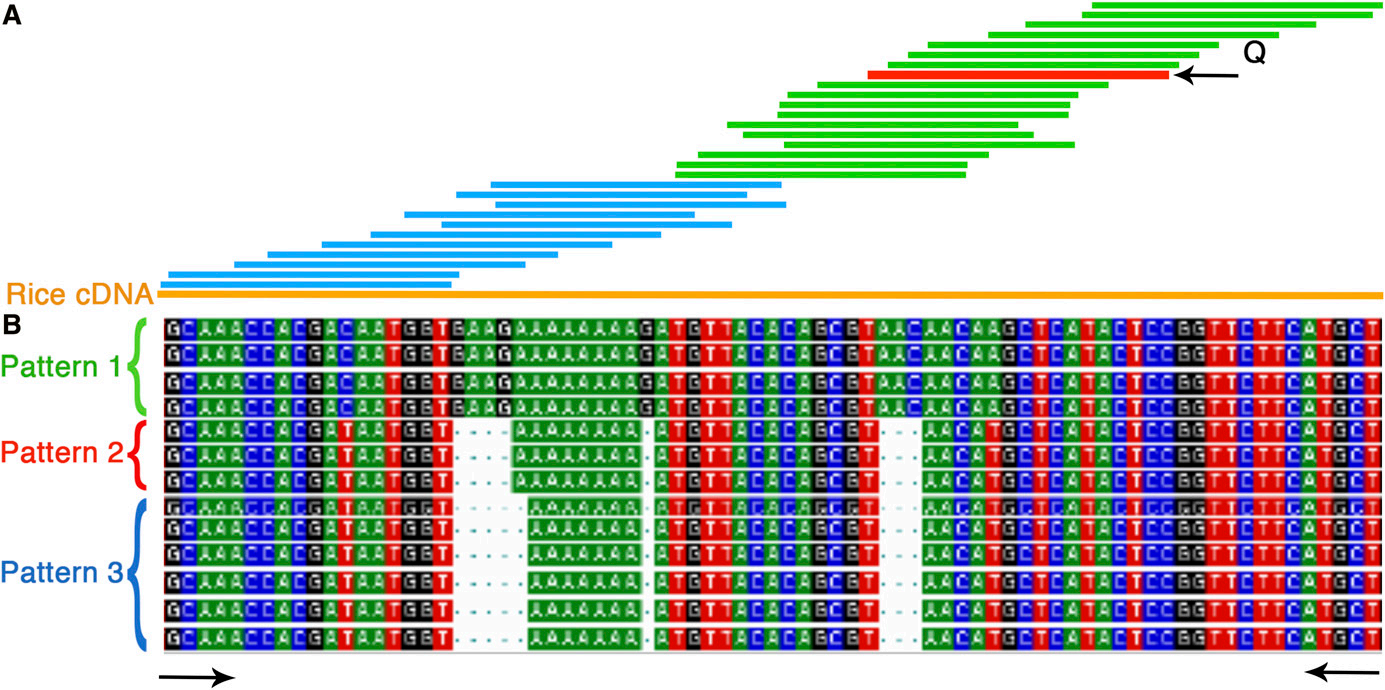
Identification of ESTs corresponding to the wheat homeologs. (A) Contig assembly of wheat homeolog ESTs corresponding to a physically mapped EST (marked red) used as a query (Q). Green bars represent the wheat ESTs identified using a query EST and blue bars denote the additional wheat ESTs identified using the corresponding rice full-length (orange bar) cDNA sequence as a bridge. (B) Sequence alignment of wheat ESTs showing different sequence patterns, each corresponding to a homeolog. ESTs showing a similar pattern are bracketed and marked by different color brackets (green, red, and blue). Black arrows mark the conserved region used to design PCR primers for the SSCP analysis. cDNA, complementary DNA; EST, expressed sequence tag; PCR, polymerase chain reaction; SSCP, single-stranded conformational polymorphism.

With the objective of studying gene expression corresponding to each of the three homeologs, EST sequences from cultivar CS were compared in detail (*Materials and Methods*). An example of the analyses and the approach to identify ESTs corresponding to each homeolog is given in Figure 1A. The EST *BE352570* (marked red, Figure 1A) was physically mapped between FL 0.45 and 0.59 on the long arm of wheat homeologous group 7 (http://wheat.pw.usda.gov/NSF/). The corresponding gene has three structural copies in the wheat genome as detected by deletion mapping. The sequence comparisons identified 28 wheat ESTs corresponding to the gene sequence (green bars). Revealed upon comparison with the corresponding rice sequence (Figure 1A, orange bar), these 28 ESTs covered 63% of the gene. A similar comparison using the full-length rice cDNA ortholog identified 18 additional wheat ESTs (Figure 1A, blue bars) extending the contig across the entire length of the gene. A similar approach was used to identify wheat ESTs for the selected 2180 wheat genes. In order to account for sequencing errors, a sequence pattern was considered to be real only if it was present in at least two of the corresponding ESTs and if the pattern was consistent along the length of the assembled contig.

Multiple alignments (Vector NTI) of the CS-specific ESTs corresponding to each of the selected genes were evaluated for the presence of distinct sequence patterns. For example, the 46 ESTs for the gene showed in Figure 1A displayed three distinct sequence patterns, showing expression of all three of the structural copies (homeologous) of the gene. This approach was used to study the expression pattern of the 2180 wheat genes. The available full-length cDNA sequence of 390 rice orthologs corresponding to these genes was used for the analysis mentioned above. Of the 2180 genes, the corresponding contigs of 1112 (51%) genes had less than five ESTs, thus, were not used for the homeologous gene expression analysis.

**Figure 2:**
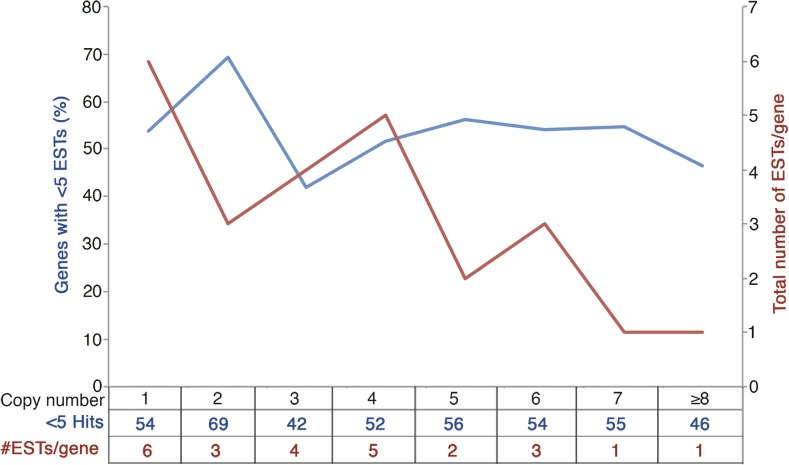
Expression pattern of genes relative to structural copy number. The *y*1-axis (blue) shows percent of genes corresponding to each structural copy number class, showing low level of expression predicted based on the proportion of genes showing < 5 ESTs (expressed sequence tags) for a gene. The *y*2-axis (red) shows the number of ESTs for each of the gene copies.

Among the analyzed genes (2180), ∼37% had the expected three structural copies and 17% had less than three copies, suggesting loss of one or two of the corresponding copies after polyploidization. Structural copies of the remaining 46% of the genes ranged from 4 to 14. The level of gene expression appears to be influenced by the number of structural copies. The proportion of the genes showing a lower level of expression (< 5 ESTs) was higher for the genes with structural copy number deviating from three (Table 1). Only 42% of the genes with three structural copies showed low levels of expression compared to 61.4% for the genes with the deviating copy number. This difference was the highest (194/280, ∼70%) for the genes with two structural copies, followed by the genes with one and five structural copies, respectively. The number of ESTs per structural copy (ESTs/copy) also showed a dramatic difference, with the least number (1) for genes with seven or more structural copies and the highest number (6) for the genes with one structural copy (Figure 2). The genes with three structural copies showed an average of four ESTs/copy. This correlation could be spurious if the number of ESTs covaries with the gene length but, as evident from Figure 2 (blue line), the number of ESTs/gene does not change significantly with the change in copy number.

**Table 1:** Expression pattern of homeologs in hexaploid wheat

Number of Structural Copies	Genes Analyzed	Number of Copies Expressed							
		7	6	5	4	3	2	1	< 5 Hits
1	95	0	0	0	0	0	0	44	51
2	280	0	0	0	0	0	68	18	194
3	813	0	0	0	0	268	172	32	341
4	359	0	0	0	46	61	52	15	185
5	293	0	0	2	26	64	32	4	165
6	166	0	0	2	18	40	11	5	90
7	64	0	0	1	6	12	7	3	35
≥ 8	110	0	1	3	9	35	10	1	51
Total	2180	0	1	8	115	488	345	111	1112

Number of structural copies was determined using the physical mapping data (see *Materials and Methods* section).

Of the genes with three structural copies, 55% expressed from all copies, 38% from two, and the remaining 7% expressed from only one of the three copies (Table 1). For the genes with two structural copies, 84% expressed from both copies whereas the remaining 16% expressed from only one. In general, the genes with more than three copies showed expression from relatively fewer copies. Only 26% of the genes with four copies showed expression from all copies and this number dropped to 1.6% for the genes with five copies. None of the genes with six or more copies showed expression from all copies (Table 1). For the genes with three or more copies, expression from three copies was the most abundant class regardless of the number of the structural copies. A small proportion of the genes were present in each category of the genes with three, two, and one copy that showed a higher number of expression patterns compared to the actual number of gene copies. This number was ∼2% for genes with three copies, ∼19% for genes with two copies, and ∼16% for the single-copy genes. None of the genes with more than three copies showed this pattern.

The ESTs used for the study were derived from 14 different tissues or developmental stages, of which 35% were obtained after exposing the plants to various stresses (Figure S1 in File S1). Analysis of the 813 genes with three structural copies for their expression in stressed *vs.* unstressed tissues, a higher proportion (48%) of the genes showing expression in the stressed tissues, were rare transcribers (< 5 ESTs) compared to those expressing in the unstressed tissues (39%). Of the genes from the stressed tissues, ∼60% expressed from all three copies compared to 52% from the unstressed tissues.

**Figure 3:**
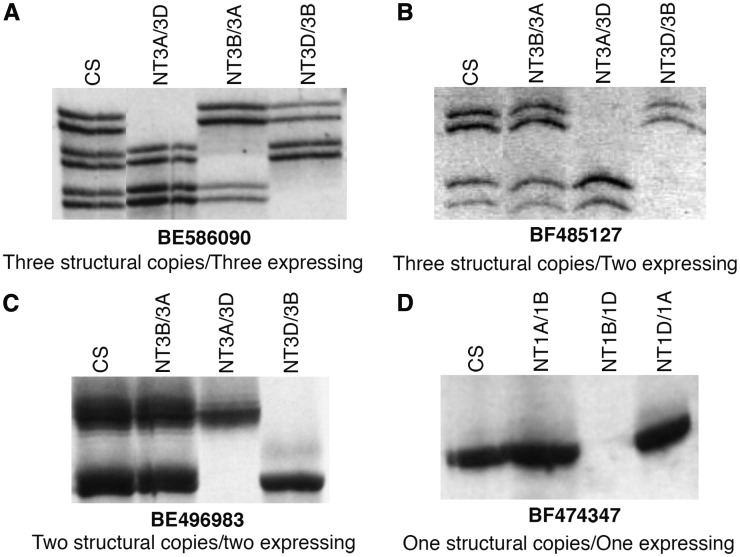
Homeologous gene expression analysis by cDNA-SSCP analysis. NTA, NTB, and NTD indicates the NT lines for the corresponding *A*, *B*, and *D* genome chromosomes. The structural copy number given under each figure is from the physical mapping of the corresponding EST by gel-blot analysis. (A–D) Varying copy number with varying number expressing is shown. cDNA, complementary DNA; CS, Chinese spring; EST, expressed sequence tag; NT, nullisomic-tetrasomic; SSCP, single-stranded conformational polymorphism.

With the objective of confirming *in silico* analysis results, 34 genes randomly selected from various structural and functional copy number classes or expression patterns were analyzed by cDNA-SSCP and standard acrylamide/urea gel analysis (*Materials and Methods*). Primers for this analysis were designed from the conserved regions flanking the sequences differentiating the different homeologs of a gene (Figure 1B). The SSCP analysis was performed on NT lines in order to identify bands corresponding to each of the three homeologs (Figure 3). Fragment bands corresponding to each of the homeologs for 16 of the 34 genes were better resolved by the cDNA-SSCP analysis, whereas the standard gels worked better for the remaining 18 genes. An example of the cDNA-SSCP analysis is given in Figure 3. The EST *BE586090* detected three structural copies, one each on the three homeologous group 3 chromosomes (http://wheat.pw.usda.gov/NSF/). The *in silico* expression analysis showed three distinct sequence patterns, suggesting that all three copies are expressing. The SSCP analysis of CS and group 3 NT lines showed three fragment bands in CS corresponding to each of the three group 3 homeologs (Figure 3A). Similarly, confirmation of the *in silico* results by the SSCP analysis for three other genes (*BF485127*, *BE496983*, and *BF474347*) with varying number of structural copies is shown in Figure 3, B–D. Likewise, the SSCP results confirmed the *in silico* results for 19 genes. Of the remaining 15, primers for two genes did not show any amplification. For 10 genes, neither of the two types of analyses was able to localize all fragment bands to their corresponding chromosomes.

Interestingly, there were some anomalies in the SSCP results where three genes (*BE517931*, *BE488792*, and *BE426712*), each expected to be expressing from all three of their structural copies, showed more than the expected number of fragment bands. The SSCP analysis for *BE517931* showed five fragment bands, of which three were not missing in any of the group 3 NT lines. The remaining two fragment bands mapped on chromosomes *3A* and *3D*. For the gene *BE488792*, the SSCP analysis showed six fragment bands, with one each mapping to *5A* and *5B* chromosomes. The group 5 NT lines did not resolve the remaining four fragment bands. Similarly, the SSCP analysis of *BE426712* showed six fragment bands, with one each mapping to *5A* and *5B*, and two mapping to the *5D* chromosome. The group 5 NT lines did not resolve the remaining two bands. More than one SSCP fragment band mapping to a chromosome may either be due to alternate splicing or highly similar, tandemly duplicated copies of the gene that were not differentiated by the RFLP analysis during the physical mapping of the corresponding ESTs.

### Tissue-specific expression of the homeologs

To study the tissue specificity of the homeologs, 268,582 wheat cultivar “CS” ESTs from 41 cDNA libraries representing 16 different tissues or developmental stages were analyzed for 220 randomly selected genes out of the 813 with three structural copies (Table 2). In total, 113 of the 220 genes expressed from all three homeologs, 85 from two, and 22 from only one in the 16-tissues/developmental stages. Expression of each of the homeologs was evaluated individually.

**Table 2:** Expression of homeologs in different wheat tissues

Tissue	Number of Homeologous Copies Expressed per Gene			Total Expressed Genes	Total Expressed Homeologs	# Expressed Homeologs/Gene	ESTs/Tissue (K)
	Three	Two	One				
Root	8	44	79	111	191	1.7	27
Shoot	3	11	36	50	67	1.3	15.5
Crown	2	12	49	63	89	1.4	15.7
Sheath	0	1	13	14	15	1	2.5
Leaf	3	10	41	54	70	1.2	23
Flag leaf	0	1	20	21	22	1	1.3
Immature spike	4	13	45	62	83	1.3	12.3
Anther	3	10	42	121	125	1	36.1
Spike HD	2	15	51	68	87	1.3	11.3
Early FL	1	10	47	99	119	1.2	47.6
Pistil HD	1	10	39	50	62	1.2	10.4
Preanthesis	4	9	57	70	87	1.2	14.3
DPA-20	7	13	46	66	93	1.4	11.3
DPA-30	3	16	35	54	76	1.4	12.6

Only the genes with three structural copies were considered for the analysis. #, number; EST, expressed sequence tag; HD, heading date; FL, flowering; DPA, days postanthesis.

**Figure 4:**
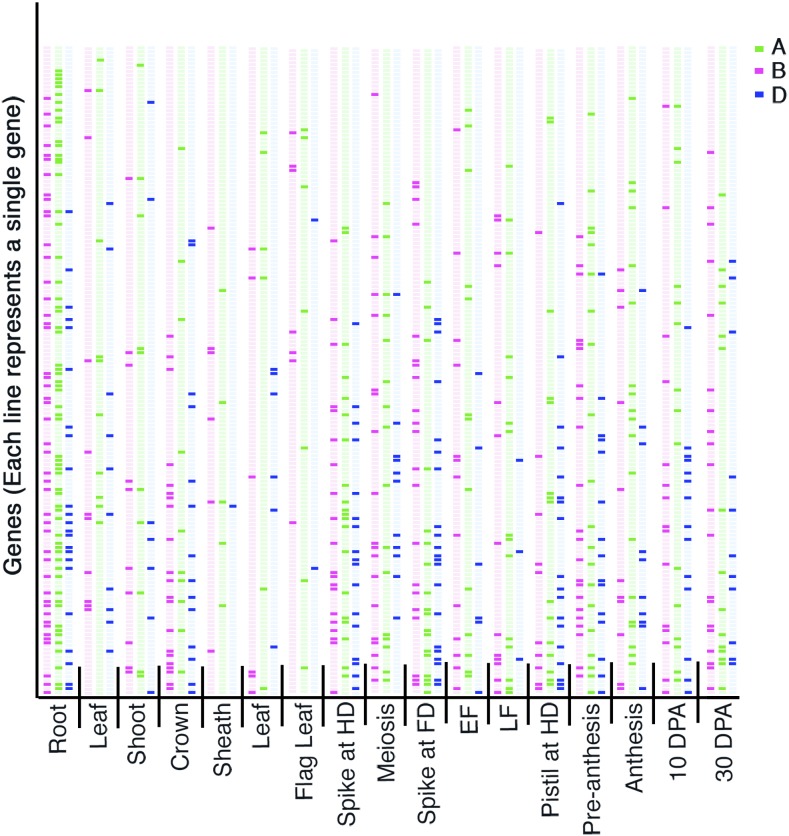
Virtual northern analysis of ESTs (expressed sequence tags) to show differential expression of homeologs during different stages of plant development. Green, pink, and blue bars correspond to three homeologs of wheat. The darker color bar indicates the presence of expression of the homeolog and the lighter bar denotes its absence. EF, early flowering; LF, later stages of flowering. Both EF and LF were collected at Feekes scale 6, so should be considered the same. FD denotes flowering date and HD heading date, whereas DPA represents days postanthesis. The EST numbers were normalized with respect to the number of actin copies.

There were major differences among tissues for the number of homeologs showing expression (Figure 4 and Table 2). The highest number of expressed homeologs/gene (1.72/3) was found in the roots and the lowest number was in the anthers (1.03/3). The number of genes showing expression was the highest in anthers/meiosis (121/220) and the lowest in leaf-sheaths (14/220). Surprisingly, the number of genes expressing in the flag leaf was also less (21/220) (Table 2), and there were significant differences among tissues for the expression level of the genes. The average number of ESTs/gene/tissue was the least in the flag leaf (1.3) and was the most in early flag leaf (47.6). The number was also higher in anthers (meiosis), roots, and leaves. For 87% of the genes, the expression pattern among homeologs was different in different tissues (Figure 4). The expression pattern among homeologs of the remaining 13% of the genes was very similar in different tissues. Unique, tissue-specific expression for at least one of the three homeologs was observed for 57% of the genes. Most of the differential expression among homeologs was observed at flowering and seed development stages with the least being in roots. Tissue specificity was more pronounced for the 22 genes that expressed from only one of the three homeologs.

**Figure 5:**
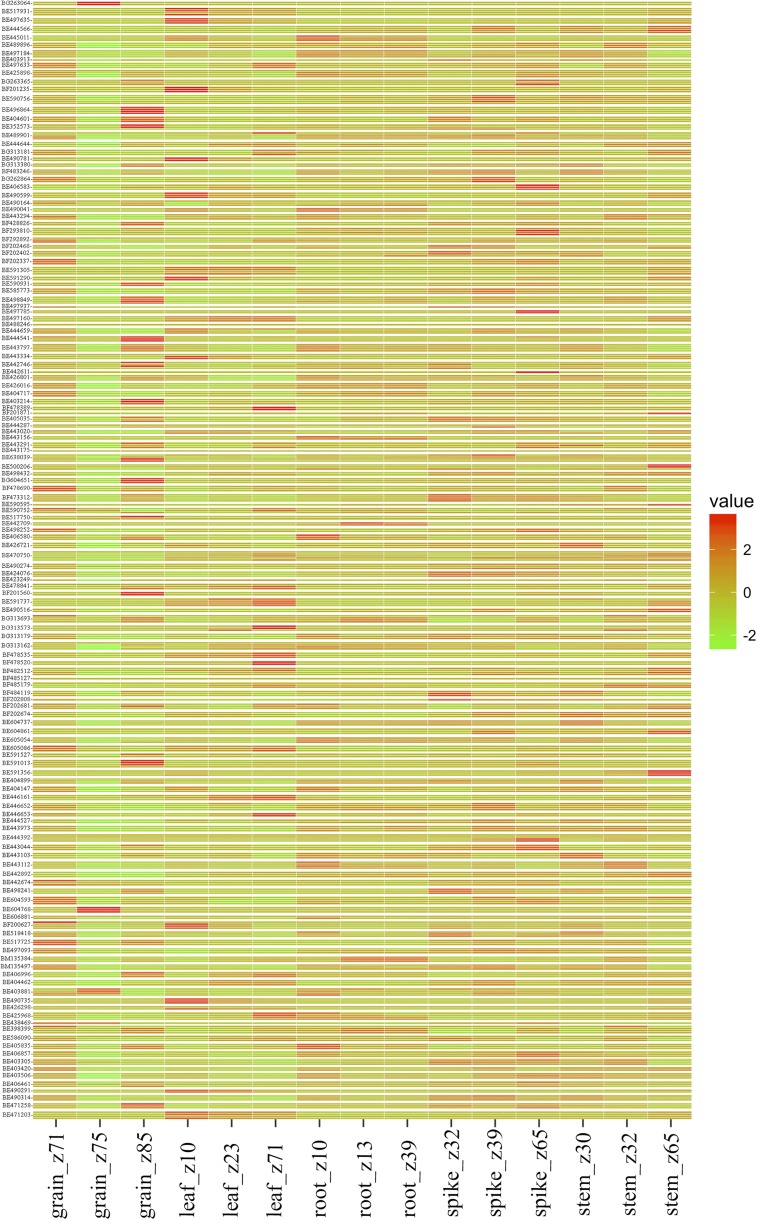
Heat map illustrating wheat EST expression patterns inferred from the RNA-seq data of multiple copies of the gene from five different developmental tissues across multiple timepoints. The expression values of the wheat ESTs with varying copy number showing differential expression patterns in three stages each of five different development tissues from different stages of the wheat life cycle (Choulet *et al.* 2014) are depicted in a heat map. Columns represent different tissue types and rows correspond to the varying copies of the ESTs indicated. The absolute expression values on a logarithmic scale are encoded by the color bar, where positive values show upregulation while the negative values show downregulation of the respective genes. EST, expressed sequence tag; RNA-seq, RNA sequencing.

Furthermore, to strengthen the tissue-specific copy differences, the RNA-seq data of multiple copies of the gene from five different developmental tissues across multiple timepoints (Choulet *et al.* 2014; Pearce *et al.* 2015) were analyzed and compared. Of the total genes analyzed, 110 (76%) showed differential expression patterns among homeologs in different tissues. In accordance with the above observation, there were major differences among tissues for the number of homeologs showing expression in addition to the expression levels. The genes showed the least expression in grain tissue collected 14 DPA, while maximum expression was observed in a spike at the meiosis stage (Figure 5). Also, the genes with three structural copies with all three expressing was the most abundant class.

**Figure 6:**
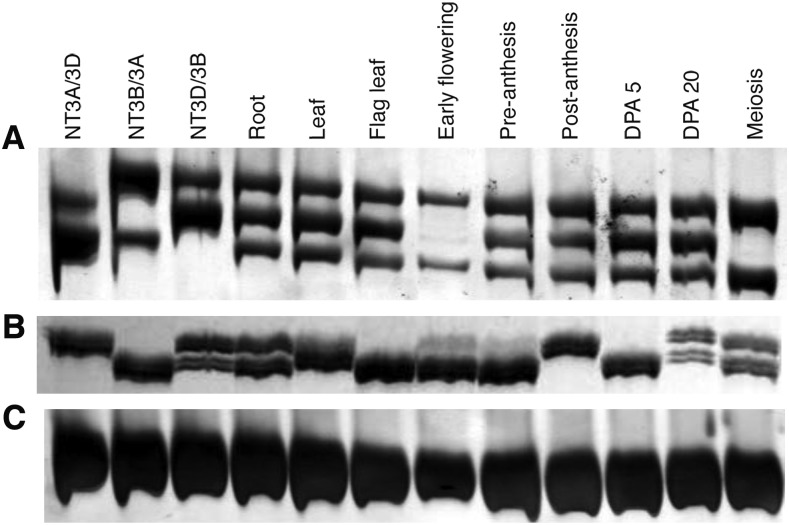
Expression analysis of homeologs in various tissues, analyzed by cDNA-SSCP analysis of various aneuploid stocks. The developmental stages included were root, leaf, flag leaf, early flowering, preanthesis, postanthesis, DPA5, DPA30, and meiosis. Expression pattern of *BE586090* (A) and *BG263365* (B) is shown relative to actin (C). Both genes have three structural copies. cDNA, complementary DNA; DPA, days postanthesis; SSCP, single-stranded conformational polymorphism.

In order to confirm *in silico*/virtual northern and RNA-seq results, tissue-specific expression of eight randomly selected genes was evaluated by the SSCP analysis of cDNA’s from nine different tissues/developmental stages of the wheat cultivar CS. The SSCP results of five of these genes (*BE586090*, *BF485127*, *BE517931*, *BG263365*, and *BE443936*) matched with that from the *in silico* analyses (virtual northern blots and RNA-seq). For example, the *in silico* analyses suggested the three homeologs for *BE586090* to be expressing in roots, leaves, DPA 5–10, and DPA 30, whereas only two of the three were predicted to express in anthers at meiosis. The SSCP analysis confirmed these results, as the *B* genome-specific fragment band was absent in the tissues at the meiotic stage but all three bands were present during other stages (Figure 6A). Similarly, for *BG263365*, two of the three structural copies were shown to express in all tissues except flag leaf, postanthesis, and DPA 5–10 (Figure 6B). Among the remaining three genes, homeolog-specific fragment bands were not resolved for *BE517750*. In the case of *BE442943*, virtual northern analysis showed all three gene copies to be expressing in the root tissue, with two of the copies expressing at DPA 30 and postanthesis. While for RNA-seq, the data was not available for this gene. The SSCP analysis showed all three gene copies to be expressing in all of the tissues analyzed, although the difference in expression level was observed among the homeologs in different tissues (Figure 6). For *BE444392*, virtual northern analysis showed expression of the three homeologs in roots only. Whereas the SSCP analysis also detected expression of the three homeologs in preanthesis, postanthesis, and DPA5. Confirming the SSCP results, the RNA-seq analysis showed expression of all of the homeologs in the late anthesis stage with some expression in roots as well. One of the three homeologs showed expression in root, leaf, and meiotic stages, but not in the flag leaf, early flowering, and DPA30 stages.

### Effect of chromosomal location on homeologous gene expression

**Figure 7:**
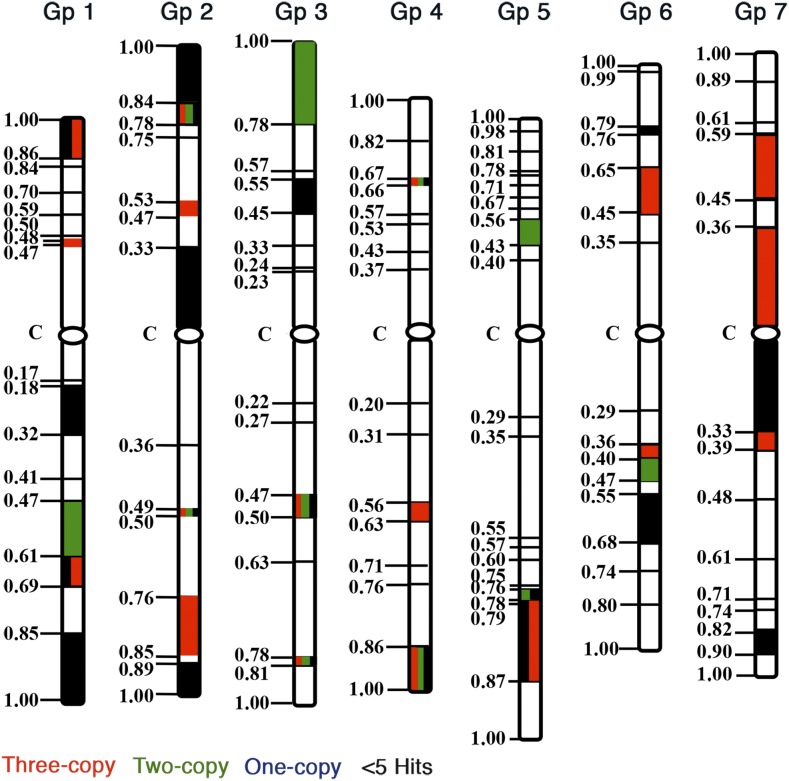
A consensus expression map of 1030 ESTs (expressed sequence tags) with three structural copies physically localized to chromosome regions bracketed by wheat deletion breakpoints. The maps were drawn using the information from Qi *et al.* (2004). Fraction length for each chromosomal region is given on the left side of the chromosomes. Statistical significance was tested at *P* < 0.05. Red colored chromosomal regions show significantly higher proportions of gene expressing from all three homeologs, green marks regions with two of the three homeologs showing expression, and the regions mark in blue show significantly a higher proportion of genes expressing from only one of the three homeologs. Chromosome regions marketed with black show a significantly higher proportion of genes with < 5 ESTs.

To study the effect of the physical location of genes on homeologous gene expression, 813 genes with three structural copies were placed on the wheat consensus physical map following the approach described by Randhawa (2004). Compared among chromosomes and after adjusting for the total number of mapped ESTs, chromosome 4 showed the highest proportion of genes with three structural copies (Figure 7 and Figure S2 in File S1). This was not because of a higher number (65) of ESTs mapping on the chromosome, as the total number of ESTs mapped on chromosome group 4 was 13.1% compared to the expected 14.3% based on a random distribution. On the other hand, group 7 chromosomes showed the lowest proportion of genes with three structural copies. The total number of mapped ESTs (14.2%) on group 7 was very close to the expected (14.3%) proportion. There were also major differences among chromosome arms for the number of three-copy genes. For example, the two arms of chromosome group 6 are similar in size but the number of three-copy genes was 2.75-times higher on the long arm.

There were also major differences, both among chromosomes as well as between chromosome arms, for the types of gene expression (Figure 7). The proportion of gene expression from all three copies was the highest in chromosome group 1 and was the lowest in group 3 chromosomes. Group 6 chromosomes on the other hand showed the highest proportion of genes expressing from two of the three copies. Group 1 chromosomes showed the lowest percentage of genes in this class.

There were significant differences among chromosome arms for the type of gene expression. About 80% of the genes present on chromosome arms 1L and 7L expressed from all three copies compared to only 28.6% for chromosome arm 3S. On the other hand, 66.7% of the genes present on chromosome arm 3S expressed from two of the three copies. The lowest number of genes expressing from two of the three copies was 11.1% for chromosome arm 1S. Similarly, the number of genes expressing from only one of the three copies was highly variable among chromosomes and chromosome arms (Figure 7).

A nonrandom distribution of gene expression pattern was also observed among various chromosomal regions (Figure 7). For example, the chromosomal segment between FL 0.47 and 0.85 on the long arm of chromosome 1 showed a significantly higher proportion of genes expressing from all three copies. Similarly, genes expressing from one of the three copies were concentrated in few chromosomal regions (Figure 7). Genes with a lower level of expression indicated by < 5 ESTs/gene were also nonrandomly distributed on the chromosomes (Figure 7).

### Dosage dependence of homeologous gene expression

**Figure 8:**
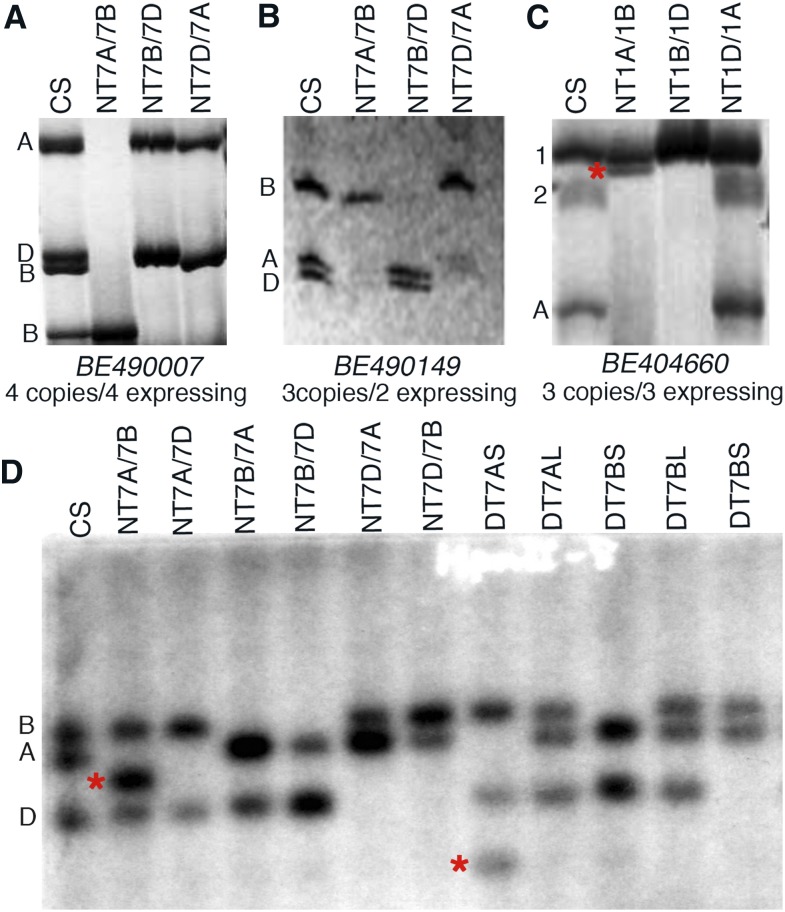
Dosage dependence of homeolog gene expression. Homeolog-specific gene expression was studied either by SSCP (single-stranded conformational polymorphism) analysis (A–C) or by using a methylation-sensitive enzyme (*Hpa*II) on genomic DNA (D). Wheat group 7 nullisomic–tetrasomic (NT) and ditelosomic (DT) lines were used to study effects of homeolog copy number change on expression or methylation pattern. The methylation pattern differences between Chinese spring (CS) and aneuploid stocks of homeologous group 7 chromosomes were studied by gel-blot analysis. The genomic DNA from CS, NT, and DT lines was digested with a methylation-sensitive enzyme and hybridized with *BE490149*. The genomic fragments identified by physical mapping are depicted as *A*, *B*, and *D* on the left of the autoradiograph. “*” indicates the fragments showing differences in methylation patterns.

The effect of copy number change on homeologous gene expression was studied via SSCP analysis of the NT lines. Seven (21%) of the 34 randomly selected genes showed a change in expression in response to a change in the homeolog copy number balance. For example, in BE490007 the physical mapping and *in silico* analyses showed four structural copies with the corresponding four SSCP bands in normal CS (Figure 8A). In the Nulli7A-Tetra7B line, the 7A band was missing as expected and expression of one of the 7B bands appeared to be more than that in CS. Surprisingly, the other 7B- and the 7D-specific bands were also missing in this line, suggesting that the expression of these two copies was silenced in response to gene copy number change in the NT line. Similarly, in the Nulli7D-Tetra7A line, one of the B-specific bands was also missing in addition to the expected absence of a D-specific band. In another example, a 7D-specific band was also missing in Nulli7A-Tetra7B (Figure 8B). The BE404660 gene has three structural copies and all three express. The SSCP analysis showed three bands in normal CS (Figure 8C), of which band #1 was present in all three NT lines. Bands #2 and #A were absent in both Nulli1A-Tetra1B as well as in Nulli1B-Tetra1D lines. An extra band (marked by “*,” Figure 8C) was present only in Nulli1A-Tetra1B. In *BF485179*, where two of the three structural copies showed expression, the 3A copy in Nuli3D-Tetra3B showed more than threefold the expression present in CS. In three other genes (*BE426712*, *BE406976*, and *BF202806*), expression of one of the homeologs was completely silenced by copy number change in the other two homeologs (data not shown).

**Figure 9:**
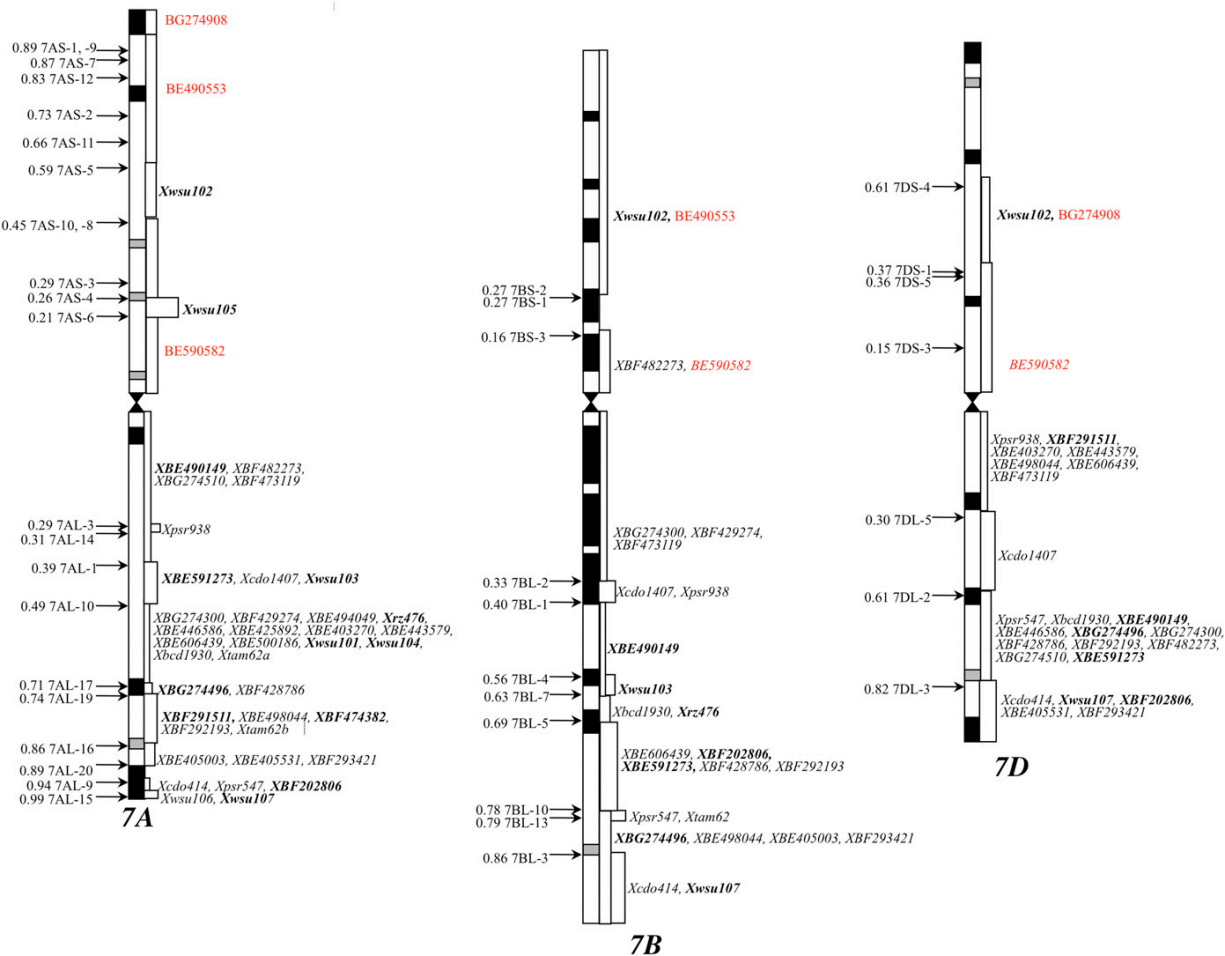
Physical map of group 7 probes. Relative chromosome length, arm ratios, and C-bands are drawn to scale. Arrows on the left of the chromosome diagrams mark the chromosome deletion breakpoints with the fraction lengths of the retained arm. The probe loci and chromosome regions paneled by the deletion breakpoints are shown on the right of the drawings. Markers in “bold” identify variable methylation patterns among the NT or DT lines when compared to normal CS. CS, Chinese spring; DT, ditelosomic; NT, nullisomic–tetrasomic.

In order to check if the dosage effect on the expression of homeologs was due to a change in DNA methylation pattern, wheat group 7 NT lines were analyzed by gel blot analysis using isochizomer restriction enzymes differing for CpG methylation sensitivity (*Hpa*II and *Msp*I). Both *Msp*I and *Hpa*II recognize the CCGG sequence for restriction, but *Hpa*II is sensitive to methylation of the second “C” in the site whereas *Msp*I is insensitive. A total of 66 wheat homeologous group 7-specific genes were used for this study, along with 17 that were specific to groups 1 or 5 that served as controls. The physical location of the group 7-specific genes is shown in Figure 9.

Of the 66 group 7 genes, 53 showed the same restriction fragment band pattern with the two enzymes, thus, no difference in methylation was observed in response to gene copy number change among group 7 NT lines. However, for the remaining 13 (20%) genes (marked “red,” Figure 9), unique fragment bands were observed with *Hpa*II in one or more of the NT or DT lines (Table 3). For *BE490149*, the B-specific fragment band in Nulli7A-Tetra7B and Nulli7A-Tetra7D indicated reduction in methylation in response to the removal of either chromosome 7A or an increased number of 7B. Furthermore, unique fragment bands were observed in the DT7AS line (Figure 8D and Table 3). Similarly, for *wsu102*, the size of the *7A*-specific fragment band was larger in Ditelo (DT) 7DS in comparison to CS, suggesting increased methylation of chromosome 7A in response to the loss of chromosome 7DS (Figure S3 in File S1 and Table 3). None of the 17 control gene probes showed any difference in methylation between CS and group 7 NT or DT lines.

**Table 3:** Methylation changes in or around genes in response to copy number changes of homeologs

Gene	Genome	CS	N7A\T7B	N7A\T7D	N7B\T7A	N7B\T7D	N7D\T7A	N7D\T7B	Dt7AS	Dt7AL	Dt7BS	Dt7BL	Dt7DS
WSU101	U	—	19	19	19	—	19, 5	—	18.5	19	7.8	—	19
WSU102	A	12	—	—	12	1	12	12	5	—	12	12	14
WSU103	B	18	20	18	—	2	18	18	12	18	—	18	18
WSU104	A	10	—	—	10	—	10	10	18	8	10	10	10
WSU105	A	18	—	—	17.8	1	18	18	—	—	18	18	18
WSU107	U	—	—	7.8	8	0	—	—	18	—	—	7.8	7.8
BF291511	A	19	—	—	—	1	17	19	7.8	19	19	19	19
BF202806	D	6	6	10	17	8	—	—	—	6	6	—	—
BF202806	B	5	5	5	6	—	5	5	6	5	—	5	5
BG274496	U	—	—	—	—	1	—	—	5	19	19	7.8	—
BE490149	B	17	12	17	—	9	17	17	—	17	—	17	17
BE490149	U	—	—	—	—	6	—	—	17	—	—	—	—
BE591273	U	—	—	7.8	—	—	10	7.8	7.8	7.8	—	—	7.8
BF474382	U	—	—	17.6	—	—	—	—	—	—	19	19	15
RZ476	U	—	18, 17.5	17.5	18	—	12	17.5	10	17.5	—	10	—

“Gene” shows the genes or ESTs that showed a difference in methylation pattern in response to copy number change; “genome” shows the genome, copy of which is showing the methylation difference; nullisomic–tetrasomic and ditelosomic lines are marked using the standard nomenclature. The numbers in the table are the sizes of the fragment bands in approximate kilobases, estimated using the size standard during the gel-blot analysis. CS, Chinese spring.

## Discussion

The effects of polyploidization on gene expression has been studied for synthetic polyploids, but the concerted fate of homeologs in natural polyploids, the interdependence of homeologous gene expression, and the corresponding mechanisms controlling these processes remain largely unexplored. Therefore, in this study, we chose wheat to address these questions as it is one of the most important cereal crops used for human consumption and, thus, any genetic insights into its genome can eventually be translated into crop improvement

Our novel *in silico* approach to identify ESTs corresponding to each of the three wheat homeologs turned out to be reliable as the results were confirmed by the SSCP analysis. The *in silico* results for the 19 genes for which all fragment bands were resolved and localized to the corresponding homeologs were confirmed by the SSCP results. The SSCP analysis of the remaining genes could not resolve all bands but the results from the polymorphic bands showed accordance between the two methods of analysis. For the three genes where the SSCP analysis showed more bands than the structural copies, this was probably due to alternate splicing or amplification from distantly related sequences. Due to a higher PCR stringency (60° annealing) the second possibility is less likely, thus, alternate splicing is a more likely explanation. Although based on the limited dataset, we argue that alternate splicing appears to be a frequent phenomenon among wheat genes.

It is difficult to study the individual expression of wheat homeologs, especially for the genes showing relatively higher levels of DNA sequence similarity. Our acrylamide/urea gel-based combination approach, using both standard and SSCP gels on wheat NT lines, proved effective as it accurately revealed the homoeologous gene expression patterns of 56% of the genes. Bands for ∼30% of the genes resolved better by the standard gels, whereas the SSCP gels worked better for the remaining 70% of the genes. Therefore, both types of gels should be run to maximally resolve the homeologous bands. All bands for the remaining 44% of the genes could not be allocated to the corresponding homeologs, probably because of comigration of bands from multiple homeologs. More sensitive methods, such as high-throughput transcriptome profiling (Liu *et al.* 2015), can be used to study homeologous-specific gene expression of this group of genes.

Here, we report that, out of the genes with three structural copies, 55% of the genes express from all three homeologs, 38% from two, and 7% from only one. Based on the analysis of 116,232 ESTs (Mochida *et al.* 2003), 26% of the wheat genes were reported to be expressing from all three homeologs, 46% from two, and 21% from one. This discrepancy is probably due to the fact that their analysis was based on ˂ one-tenth of the ESTs that we used for our analysis and that the structural copy number information was not used. Accurate estimation of the number of expressed homeologs/gene cannot be determined without knowing the actual number of structural copies of the corresponding genes along with a comprehensive set of ESTs from different wheat tissues. Also, their analysis was based on ESTs and not the full-length gene sequences, thus, was prone to error particularly for larger sized genes where two nonoverlapping EST contigs corresponding to a gene would be counted twice. Our analysis was based on full-length gene sequences constructed using rice full-length cDNA sequences as a “bridge.” This approach was successful in identifying full-length wheat gene sequences.

One of the key finding of our study is that homeologs for at least 87% of the wheat genes have developed different gene expression patterns during the course of polyploidization. Of the 198 genes with three structural copies, the expression patterns of homeologs of 173 (87%) differed in different tissues. The level of sequence conservation among homeologs of 87% of the genes was not significantly different from that of the remaining 13%. Any change in expression pattern in different tissues may result in a novel function, thus, could be an important force to evolve new gene functions. Among tissues, stages after flower initiation showed homeolog-specific expression whereas vegetative stages usually showed expression from multiple homeologs (Table 2).

We observed that genes with three structural copies showed the most consistent level of expression. Any deviation in copy number showed an overall reduction in the expression level. The number of ESTs/homeologs for the genes with three structural copies was four. The corresponding number for the genes with only one structural copy was six and for the genes with seven structural copies was one. Any deviation in genomic copy number leads to an overall reduction in expression at the gene level. At the gene level, the number of ESTs for the genes with one structural copy was six and for the genes with seven gene copies was seven. These numbers were significantly lower than 12 ESTs/gene found for the genes with three structural copies. A balance of homeolog expression was observed, as any deviation in copy number of the gene resulted in altered expression levels. These conclusions further strengthened the effect of dosage on gene expression.

Uneven distribution of genes is a common feature of eukaryotes, although the unevenness is more pronounced in species with larger genomes (Gill *et al.* 1993; Sidhu and Gill 2005; Schnable *et al.* 2011) that contain only a small fraction of the genic regions and large amounts of repetitive elements. Distribution of recombination is also highly uneven in eukaryotes and the recombination is usually limited to the genic regions (Gill *et al.* 1993; Sidhu and Gill 2005; Schnable *et al.* 2011). Additionally, even regions within the gene-rich regions show a lot of variation with respect to the recombination frequency. These observations suggest that different chromosomal regions are structurally and functionally distinct. In the present study, we have identified several chromosomal regions/arms showing uneven distribution of various expression patterns (Figure 7 and Figure S2 in File S1). In general, pericentromeric and proximal chromosomal regions known to contain “housekeeping” genes favored the expression of all three homeologs. On the other hand, distal regions housed genes expressing from one or two of the three homeologs. The level of gene expression was also nonrandomly distributed on the chromosomes. The number of genes with low levels of expression (< 5 ESTs/gene) was disproportionately higher in chromosomal regions 1S-0.86-1.00, 1L-0.17-0.18, 4S-0.66-0.67, 6S-0.99-1.00, and 7S-0.59-0.61, whereas the number of genes with abundant expression was higher in the regions 1S-0.50-0.59, 1L-0.69-0.61, 2S-0.47-0.53, 2L-0.50-0.49, 2L-0.89-0.85, 3S-0.23-0.24, 3L-0.78-0.81, 4S-0.37-0.43, 4L-0.86-1.00, 5L-0.75-0.76, 6S-0.35-0.45, 6L-0.36-0.40, and 6L-0.74-0.80. We have evidently shown that the chromosomal arm and subarm locations, apart from having an effect on recombination, also impact the gene expression pattern of duplicated genes.

Independently studied by methylation differences and SSCP-based expression analysis, we showed that ∼20% of the genes show expression differences in response to gene copy balance change of homeologs in NT lines. Of the genes that showed methylation differences, 54% showed a novel band in one or more of the aneuploids with 31% of the bands being smaller than the expected band (loss of methylation), whereas bigger (gain of methylation) than expected bands were observed for the remaining 23% of the genes. These observations suggested that the changes in DNA methylation patterns are one of the factors determining the effect of dosage on the expression of homeologs.

## Supplementary Material

Supplemental material is available online at www.g3journal.org/lookup/suppl/doi:10.1534/g3.116.038711/-/DC1.

## References

[bib1] Adams K. L. Wendel J. F., 2005 Polyploidy and genome evolution in plants. Curr. Opin. Plant Biol. 8: 135–141. 15752992 10.1016/j.pbi.2005.01.001

[bib2] Adams K. L. Cronn R. Percifield R. Wendel J. F., 2003 Genes duplicated by polyploidy show unequal contributions to the transcriptome and organ-specific reciprocal silencing. Proc. Natl. Acad. Sci. USA 100: 4649–4654. 12665616 10.1073/pnas.0630618100 PMC153610

[bib3] Akhunova A. R. Matniyazov R. T. Liang H. Akhunov E. D., 2010 Homoeolog-specific transcriptional bias in allopolyploid wheat. BMC Genomics 11: 1–16. 20849627 10.1186/1471-2164-11-505 PMC2997001

[bib4] Arnaiz O. Goût J. F. Bétermier M. Bouhouche K. Cohen J., 2010 Gene expression in a paleopolyploid: a transcriptome resource for the ciliate Paramecium tetraurelia. BMC Genomics 11: 547. 20932287 10.1186/1471-2164-11-547 PMC3091696

[bib5] Aury J. M. Jaillon O. Laudet V. Robinson-Rechavik M., 2006 Gene loss and evolutionary rates following whole-genome duplication in teleost fishes. Mol. Biol. Evol. 23: 1808–1816. 16809621 10.1093/molbev/msl049

[bib6] Blanc G. Wolfe K. H., 2004 Widespread paleopolyploidy in model plant species inferred from age distributions of duplicate genes. Plant Cell 16: 1667–1678. 15208399 10.1105/tpc.021345 PMC514152

[bib7] Bombarely A. Coate J. E. Doyle J. J., 2014 Mining transcriptomic data to study the origins and evolution of a plant allopolyploid complex. PeerJ 2: e391. 24883252 10.7717/peerj.391 PMC4034613

[bib8] Bottley A. Xia G. M. Koebner R. M. D., 2006 Homoeologous gene silencing in hexaploid wheat. Plant J. 47: 897–906. 16899084 10.1111/j.1365-313X.2006.02841.x

[bib9] Buggs R. J. A. Elliott N. M. Zhang L. Koh J. Viccini L. F., 2010 Tissue‐specific silencing of homoeologs in natural populations of the recent allopolyploid *Tragopogon mirus*. New Phytol. 186: 175–183. 20409177 10.1111/j.1469-8137.2010.03205.x

[bib10] Buggs R. J. A. Soltis P. S. Soltis D. E., 2011 Biosystematic relationships and the formation of polyploids. Taxon 60: 324–332.

[bib11] Chaudhary B. Flagel L. Stupar R. M. Udall J. A. Verma N., 2009 Reciprocal silencing, transcriptional bias and functional divergence of homeologs in polyploid cotton (gossypium). Genetics 182: 503–517. 19363125 10.1534/genetics.109.102608 PMC2691759

[bib12] Choulet F. Alberti A. Theil S. Glover N. Barbe V., 2014 Structural and functional partitioning of bread wheat chromosome 3B. Science 345: 1249721. 25035497 10.1126/science.1249721

[bib13] Comai L., 2005 The advantages and disadvantages of being polyploid. Nat. Rev. Genet. 6: 836–846. 16304599 10.1038/nrg1711

[bib14] Conley E. J., 2004 A 2600-locus chromosome bin map of wheat homoeologous group 2 reveals interstitial gene-rich islands and colinearity with rice. Genetics 168: 625–637. 15514040 10.1534/genetics.104.034801 PMC1448822

[bib15] Cronn R. C. Adams K. L., 2003 Quantitative analysis of transcript accumulation from genes duplicated by polyploidy using cDNA-SSCP. Biotechniques 34: 726–734. 12703297 10.2144/03344st01

[bib16] Dilbirligi M. Erayman M. Sandhu D. Sidhu D. Gill K. S., 2004 Identification of wheat chromosomal regions containing expressed resistance genes. Genetics 166: 461–481. 15020436 10.1534/genetics.166.1.461 PMC1470719

[bib17] Doležel J. Vrána J. Šafář J. Bartoš J. Kubaláková M., 2012 Chromosomes in the flow to simplify genome analysis. Funct. Integr. Genomics 12: 397–416. 22895700 10.1007/s10142-012-0293-0 PMC3431466

[bib18] Endo T. R., 1988 Induction of chromosomal structural changes by a chromosome of *Aegilops cylindrica* L. in common wheat. J. Hered. 79: 366–370. 10.1093/oxfordjournals.jhered.a110529 31581766

[bib19] Endo T. R. Gill B. S., 1996 The deletion stocks of common wheat. J. Hered. 87: 295–307.

[bib20] Erayman M. Sandhu D. Sidhu D. Dilbirligi M. Baenziger P. S., 2004 Demarcating the gene-rich regions of the wheat genome. Nucleic Acids Res. 32: 3546–3565. 15240829 10.1093/nar/gkh639 PMC484162

[bib21] Feldman M. Mello-Sampayo T. Sears E. R., 1966 Somatic association in *Triticum aestivum*. Proc. Natl. Acad. Sci. USA 56: 1192–1199. 5230145 10.1073/pnas.56.4.1192 PMC220038

[bib22] Feldman M. Liu B. Segal G. Abbo S. Levy A. A., 1997 Rapid elimination of low-copy DNA sequences in polyploid wheat: a possible mechanism for differentiation of homoeologous chromosomes. Genetics 147: 1381–1387. 9383078 10.1093/genetics/147.3.1381 PMC1208259

[bib23] Fielden M. R. Matthews J. B. Fertuck K. C. Halgren R. G. Zacharewski T. R., 2002 In silico approaches to mechanistic and predictive toxicology: an introduction to bioinformatics for toxicologists. Crit. Rev. Toxicol. 32: 67–112. 11951993 10.1080/20024091064183

[bib24] Gill K. S. Gill B. S., 1994 Mapping in the realm of polyploidy - the wheat model. BioEssays 16: 841–846.

[bib25] Gill K. S. Gill B. S. Endo T. R., 1993 A chromosome region-specific mapping strategy reveals gene-rich telomeric ends in wheat. Chromosoma 102: 374–381.

[bib26] Grant V., 1981 *Plant Speciation*, Ed. 2 Columbia University Press, New York.

[bib27] Groszmann M. Paicu T. Alvarez J. P. Swain S. M. Smyth D. R., 2011 *Spatula* and *Alcatraz* are partially redundant, functionally diverging *Bhlh* genes required for Arabidopsis gynoecium and fruit development. Plant J. 68: 816–829. 21801252 10.1111/j.1365-313X.2011.04732.x

[bib28] Grusz A. L. Windham M. D. Pryer K. M., 2009 Deciphering the origins of apomictic polyploids in the *Cheilanthes yavapensis* complex (Pteridaceae). Am. J. Bot. 96: 1636–1645. 21622350 10.3732/ajb.0900019

[bib29] Gu Z. Nicolae D. Lu H. H. S. Li W. H., 2002 Rapid divergence in expression between duplicate genes inferred from microarray data. Trends Genet. 18: 609–613. 12446139 10.1016/s0168-9525(02)02837-8

[bib30] Hossain K. G., 2004 A chromosome bin map of 2148 expressed sequence tag loci of wheat homoeologous group 7. Genetics 168: 687–699. 15514045 10.1534/genetics.104.034850 PMC1448827

[bib31] Huang S. Sirikhachornkit A. Su X. Faris J. Gill B., 2002 Genes encoding plastid acetyl-CoA carboxylase and 3-phosphoglycerate kinase of the Triticum/Aegilops complex and the evolutionary history of polyploid wheat. Proc. Natl. Acad. Sci. USA 99: 8133–8138. 12060759 10.1073/pnas.072223799 PMC123033

[bib32] Jeffrey Chen Z. Wang J. Tian L. Lee H. S. Wang J. J., 2004 The development of an Arabidopsis model system for genome-wide analysis of polyploidy effects. Biol. J. Linn. Soc. Lond. 82: 689–700. 18079994 10.1111/j.1095-8312.2004.00351.x PMC2136415

[bib33] Kashkush K. Feldman M. Levy A. A., 2003 Transcriptional activation of retrotransposons alters the expression of adjacent genes in wheat. Nat. Genet. 33: 102–106. 12483211 10.1038/ng1063

[bib34] Keller B. Grote K. Adamski J., 2006 In silico Northern blot, an automated method to determine expression patterns from EST databases, reveals tissue specificity of murine 17beta-hydroxysteroid dehydrogenase type 11. Mol. Cell. Endocrinol. 248: 242–245. 16406283 10.1016/j.mce.2005.11.033

[bib35] Kellogg E. A., 2015 Genome sequencing: long reads for a short plant. Nat. Plants 1: 15169. 27251714 10.1038/nplants.2015.169

[bib36] Kihara H., 1944 Discovery of the DD-analyser, one of the ancestors of vulgare wheats. Agric. Hortic. 19: 889–890.

[bib37] Koh J. Soltis P. S. Soltis D. E., 2010 Homeolog loss and expression changes in natural populations of the recently and repeatedly formed allotetraploid *Tragopogon mirus* (Asteraceae). BMC Genomics 11: 1–16. 20141639 10.1186/1471-2164-11-97 PMC2829515

[bib38] Large E. C., 1954 Growth stages in cereals illustration of the Feekes scale. Plant Pathol. 3: 128–129.

[bib39] Li J. T. Hou G.-Y. Kong X. F. Li C.-Y. Zeng J.-M., 2015 The fate of recent duplicated genes following a fourth-round whole genome duplication in a tetraploid fish, common carp (*Cyprinus carpio*). Sci. Rep. 5: 8199. 25645996 10.1038/srep08199 PMC4314655

[bib40] Liu Z. Xin M. Qin J. Peng H. Ni Z., 2015 Temporal transcriptome profiling reveals expression partitioning of homeologous genes contributing to heat and drought acclimation in wheat (*Triticum aestivum* L.). BMC Plant Biol. 15: 152. 26092253 10.1186/s12870-015-0511-8 PMC4474349

[bib41] Lynch M. Conery J. S., 2000 The evolutionary fate and consequences of duplicate genes. Science 290: 1151–1155. 11073452 10.1126/science.290.5494.1151

[bib42] Masterson J., 1994 Stomatal size in fossil plants: evidence for polyploidy in majority of angiosperms. Science 264: 421–424. 17836906 10.1126/science.264.5157.421

[bib43] McFadden E. Sears E., 1946 The origin of Triticum spelta and its free-threshing hexaploid relatives. J. Hered. 37: 81–107. 20985728 10.1093/oxfordjournals.jhered.a105590

[bib44] Miftahudin K. Ross X. F. Ma A. A. Mahmoud J. Layton, 2004 Analysis of expressed sequence tag loci on wheat chromosome group 4. Genetics 168: 651–663. 15514042 10.1534/genetics.104.034827 PMC1448824

[bib45] Ming R. Wai C. M., 2015 Assembling allopolyploid genomes: no longer formidable. Genome Biol. 16: 1. 25723730 10.1186/s13059-015-0585-5 PMC4312463

[bib46] Mochida K. Yamazaki Y. Ogihara Y., 2003 Discrimination of homoeologous gene expression in hexaploid wheat by SNP analysis of contigs grouped from a large number of expressed sequence tags. Molecular genetics and genomics. Mol. Genet. Genomics 270: 371–377. 14595557 10.1007/s00438-003-0939-7

[bib47] Munkvold J. D., 2004 Group 3 chromosome bin maps of wheat and their relationship to rice chromosome 1. Genetics 168: 639–650. 15514041 10.1534/genetics.104.034819 PMC1448823

[bib48] Mutti J. S. Sandhu D. Sidhu D. Gill K. S., 2010 Dynamic nature of a wheat centromere with a functional gene. Mol. Breed. 26: 177–187.

[bib49] Nomura T. Ishihara A. Yanagita R. C. Endo T. R. Iwamura H., 2005 Three genomes differentially contribute to the biosynthesis of benzoxazinones in hexaploid wheat. Proc. Natl. Acad. Sci. USA 102: 16490–16495. 16260753 10.1073/pnas.0505156102 PMC1283429

[bib50] Pearce S. Vazquez-Gross H. Herin S. Y. Hane D. Wang Y., 2015 WheatExp: an RNA-seq expression database for polyploid wheat. BMC Plant Biol. 15: 1. 26705106 10.1186/s12870-015-0692-1 PMC4690421

[bib51] Peng J. H., 2004 Chromosome bin map of expressed sequence tags in homoeologous group 1 of hexaploid wheat and homoeology with rice and *Arabidopsis*. Genetics 168: 609–623. 15514039 10.1534/genetics.104.034793 PMC1448821

[bib52] Pfeifer M. Kugler K. G. Sandve S. R. Zhan B. Rudi H., 2014 Genome interplay in the grain transcriptome of hexaploid bread wheat. Science 345: 1250091. 25035498 10.1126/science.1250091

[bib53] Qi L. L. Echalier B. Chao S. Lazo G. R. Butler G. E., 2004 A chromosome bin map of 16,000 expressed sequence tag loci and distribution of genes among the three genomes of polyploid wheat. Genetics 168: 701–712. 15514046 10.1534/genetics.104.034868 PMC1448828

[bib54] Randhawa H. S., 2004 Deletion mapping of homoeologous group 6-specific wheat expressed sequence tags. Genetics 168: 677–686. 15514044 10.1534/genetics.104.034843 PMC1448826

[bib55] Roulin A. Auer P. L. Libault M. Schlueter J. Farmer A., 2013 The fate of duplicated genes in a polyploid plant genome. Plant J. 73: 143–153. 22974547 10.1111/tpj.12026

[bib56] Salmon A. Ainouche M. L. Wendel J. F., 2005 Genetic and epigenetic consequences of recent hybridization and polyploidy in Spartina (Poaceae). Mol. Ecol. 14: 1163–1175. 15773943 10.1111/j.1365-294X.2005.02488.x

[bib57] Sandhu D. Champoux J. A. Bondareva S. N. Gill K. S., 2001 Identification and physical localization of useful genes and markers to a major gene-rich region on wheat group 1S chromosomes. Genetics 157: 1735–1747. 11290727 10.1093/genetics/157.4.1735 PMC1461613

[bib58] Sasanuma T. Miyashita N. Tsunewaki K., 1996 Wheat phylogeny determined by RFLP analysis of nuclear DNA. 3. Intra- and interspecific variations of five *Aegilops* Sitopsis species. Theor. Appl. Genet. 92: 928–934. 24166619 10.1007/BF00224032

[bib59] Schnable J. C. Springer N. M. Freeling M., 2011 Differentiation of the maize subgenomes by genome dominance and both ancient and ongoing gene loss. Proc. Natl. Acad. Sci. USA 108: 4069–4074. 21368132 10.1073/pnas.1101368108 PMC3053962

[bib60] Sears E. R., 1954 The aneuploids of common wheat. Mo. Agric. Exp. Stn. Res. Bull. 572: 1–58.

[bib61] Sears, E. R., and L. M. S. Sears 1978 The use of telocentric chromosomes of common wheat. Proceedings of the Fifth International Wheat Genetics Symposium, February, 1978, New Delhi.

[bib62] Sémon M. Wolfe K. H., 2008 Preferential subfunctionalization of slow-evolving genes after allopolyploidization in *Xenopus laevis*. Proc. Natl. Acad. Sci. USA 105: 8333–8338. 18541921 10.1073/pnas.0708705105 PMC2448837

[bib63] Shaked H. Kashkush K. Ozkan H. Feldman M. Levy A. A., 2001 Sequence elimination and cytosine methylation are rapid and reproducible responses of the genome to wide hybridization and allopolyploidy in wheat. Plant Cell 13: 1749–1759. 11487690 10.1105/TPC.010083 PMC139131

[bib64] Sidhu D. Gill K. S., 2005 Distribution of genes and recombination in wheat and other eukaryotes. Plant Cell Tissue Organ Cult. 79: 257–270.

[bib65] Song K. Lu P. Tang K. Osborn T. C., 1995 Rapid genome change in synthetic polyploids of brassica and its implications for polyploid evolution. Proc. Natl. Acad. Sci. USA 92: 7719–7723. 7644483 10.1073/pnas.92.17.7719 PMC41217

[bib66] Stebbins G. L., 1950 *Variation and Evolution in Plants*. Columbia University Press, New York.

[bib67] Tate J. A. Ni Z. Scheen A. C. Koh J. Gilbert C. A., 2006 Evolution and expression of homeologous loci in *Tragopogon miscellus* (Asteraceae), a recent and reciprocally formed allopolyploid. Genetics 173: 1599–1611. 16648586 10.1534/genetics.106.057646 PMC1526671

[bib68] Thomas B. C. Pedersen B. Freeling M., 2006 Following tetraploidy in an Arabidopsis ancestor, genes were removed preferentially from one homeolog leaving clusters enriched in dose-sensitive genes. Genome Res. 16: 934–946. 16760422 10.1101/gr.4708406 PMC1484460

[bib69] Udall J. A. Wendel J. F., 2006 Polyploidy and crop improvement. Crop Sci. 46: 3–14.

[bib70] Venter J. C. Adams M. D. Myers E. W. Li P. W. Mural R. J., 2001 The sequence of the human genome. Science 291: 1304–1351. 11181995 10.1126/science.1058040

[bib71] Wendel J. F. Cronn R. C., 2003 Polyploidy and the evolutionary history of cotton. Adv. Agron. 78: 139–186.

[bib72] Wolfe K. H. Shields D. C., 1997 Molecular evidence for an ancient duplication of the entire yeast genome. Nature 387: 708–713. 9192896 10.1038/42711

[bib73] Yoo M. J. Wendel J. F., 2014 Comparative evolutionary and developmental dynamics of the cotton (*Gossypium hirsutum*) fiber transcriptome. PLoS Genet. 10: e1004073. 24391525 10.1371/journal.pgen.1004073 PMC3879233

[bib74] Yoo M. J. Szadkowski E. Wendel J. F., 2013 Homoeolog expression bias and expression level dominance in allopolyploid cotton. Heredity 110: 171–180. 23169565 10.1038/hdy.2012.94 PMC3554454

