# Supplementary material for: Evolution of Gene Expression Balance Among Homeologs of Natural Polyploids
Source: G3 (Bethesda). 2017 Feb 13;7(4):1225–37. doi: 10.1534/g3.116.038711 (PMC5386871; doi:10.1534/g3.116.038711)
Supplement: Supplementary file 1 [file 1225file002.pdf]

Fig. S1

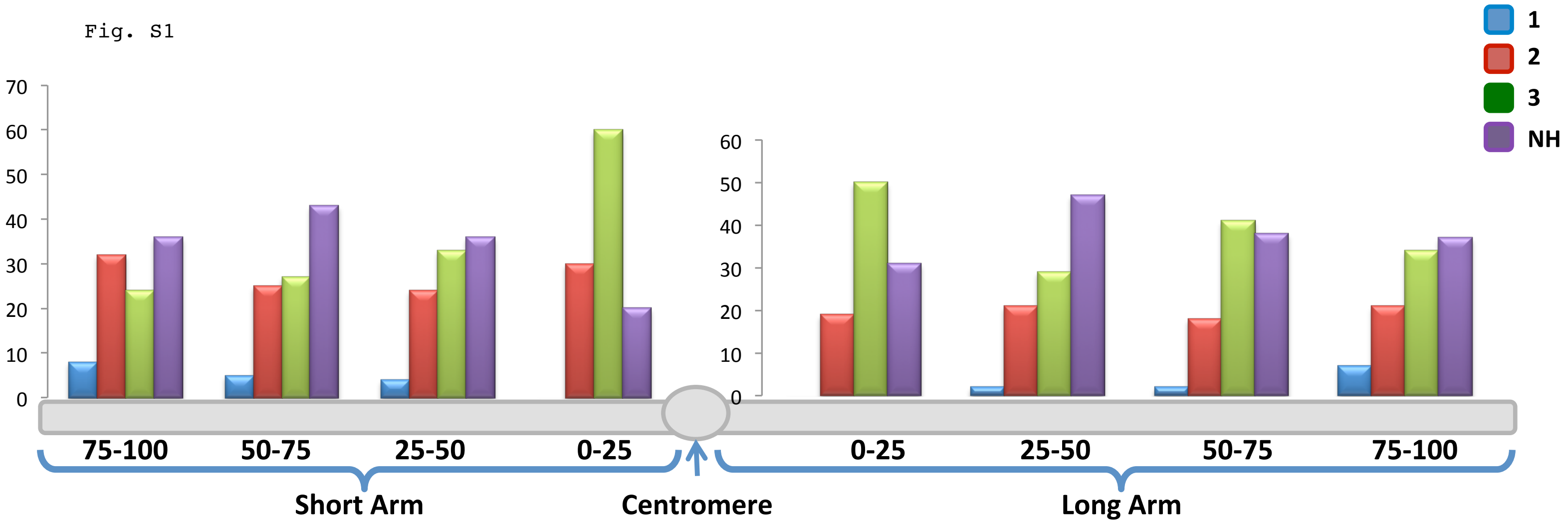

Short Arm						
**# of Patterns**	**1**	**2**	**3**	**4**	**NH**	**Total**
0-25	x	3	6	x	2	**10**
Percentage	0	30	60	0	20	
25-50	4	24	33	2	36	**99**
Percentage	4	24	33	2	36	
50-75	4	19	21	x	33	**77**
Percentage	5	25	27	0	43	
75-100	5	21	16	x	24	**66**
Percentage	8	32	24	0	36	

Long Arm						
**# of Patterns**	**1**	**2**	**3**	**4**	**NH**	**Total**
0-25	x	7	18	x	11	**36**
Percentage	0	19	50	0	31	
25-50	4	35	49	2	80	**170**
Percentage	2	21	29	1	47	
50-75	2	15	34	1	32	**84**
Percentage	2	18	41	1	38	
75-100	11	34	54	2	58	**159**
Percentage	7	21	34	1	37	

Supplementary figure 1. Expression pattern summary of genes with three structural copies along the chromosome. Each arm of a hypothetical chromosome was divided into four quarters and the expression pattern was averaged over all chromosomes. The 1, 2, and 3 correspond to the number of copies expressing for the genes. 'NH' represents genes with lower level of expression determined from the number of ESTs. Only the genes with three structural copies were used for the analysis.

Fig. S2

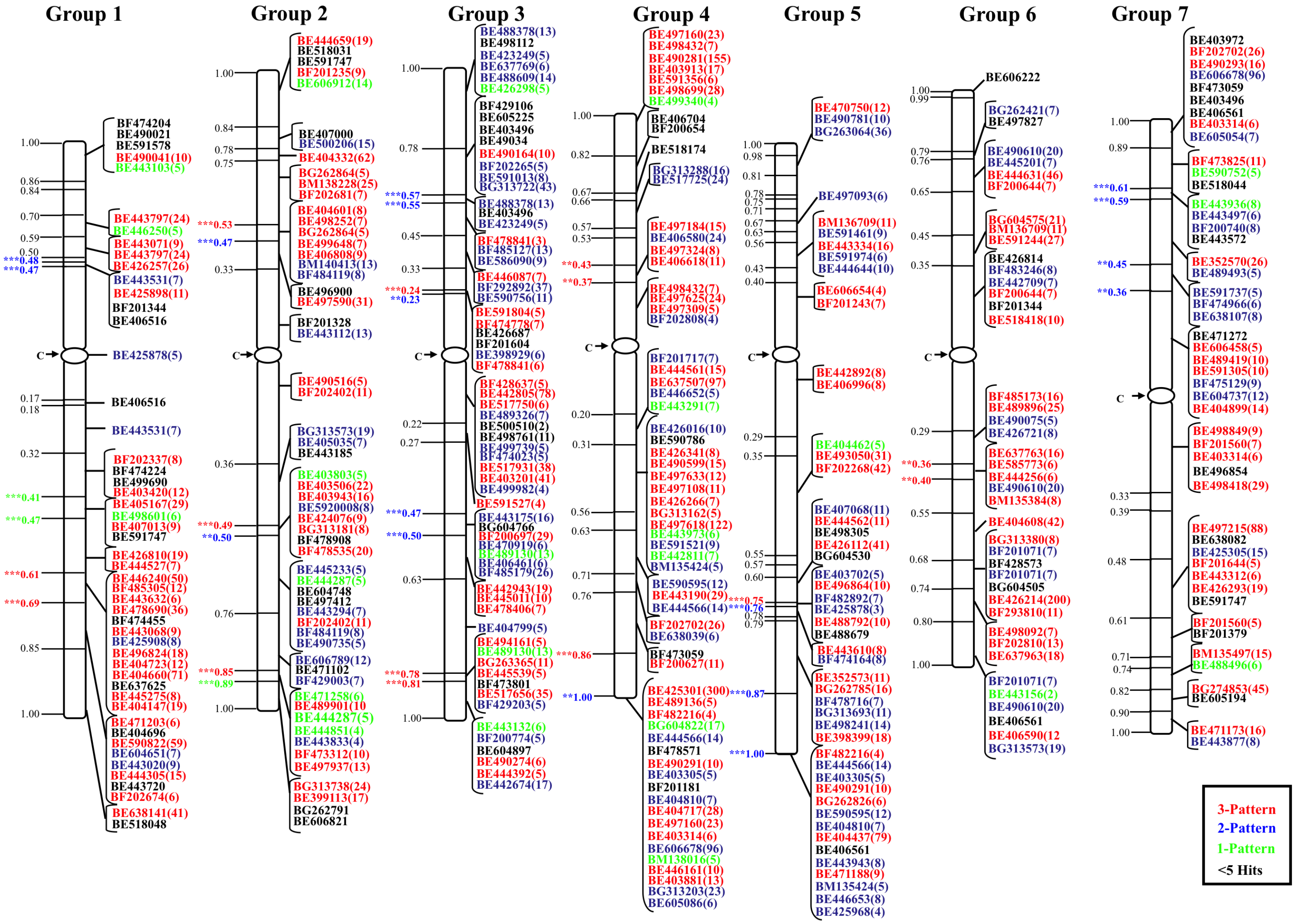

Supplementary figure 2. Distribution of expression pattern on wheat chromosomes. A consensus expression map of 1030 ESTs with three structural copies physically localized to chromosome regions bracketed by wheat deletion breakpoints. The maps were drawn using the information from Qi et al. 2004. Fraction length for each chromosomal regions is given on the left side of the chromosomes and the genes are given on the right. Statistical significance was tested at p <0.05. The genes showing expression from all three copies are marked 'Red', from two of the three copies are marked 'Blue' and the ones expressing from one of the three copies are marked 'Green'. Genes marked 'Black' are the genes showing less than 5 ESTs.

Fig. S3

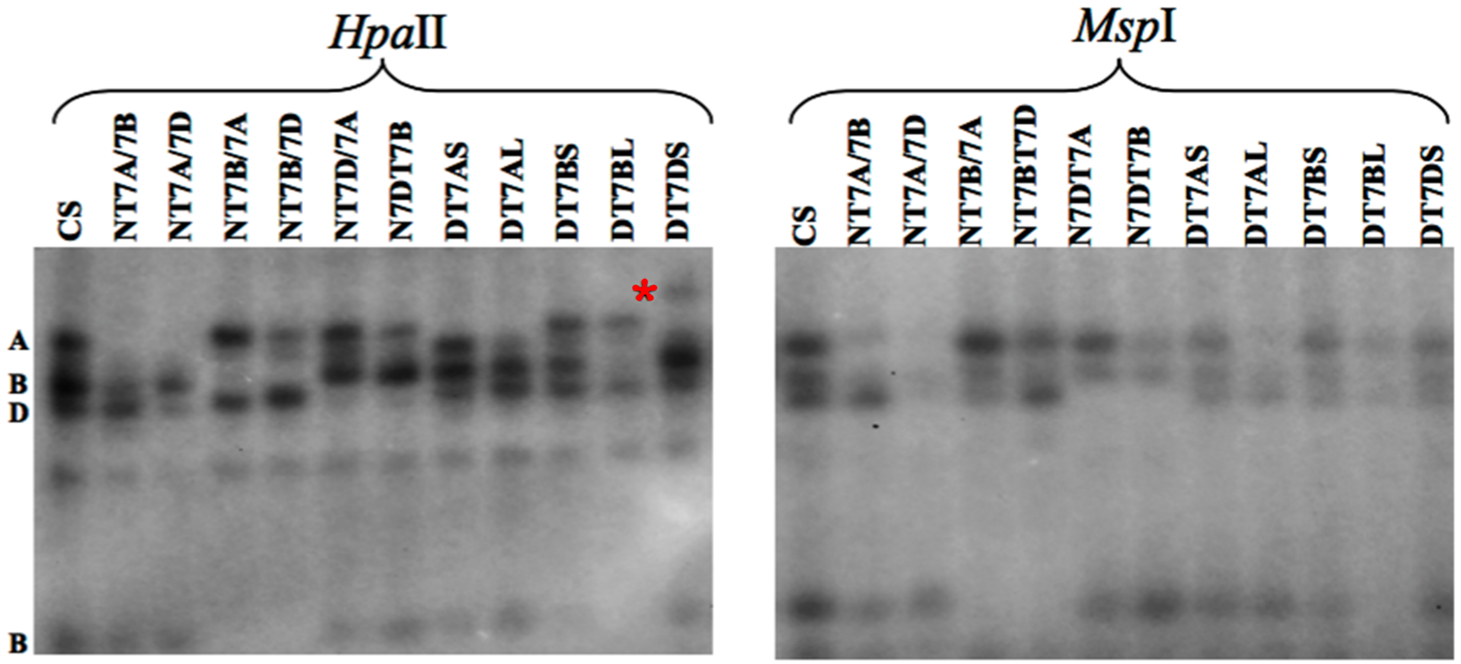

Supplementary Figure 3. Methylation pattern between CS and aneuploid stocks of homoeologous group 7 chromosomes. The autoradiographs of southern hybridization of genomic DNA of normal 'Chinese spring' (CS), nullisomic-tetrasomic (NT), and ditelosomic (DT) lines for group 7 chromosomes. The genomic DNA from CS, NT and DT lines was digested with a methylation-sensitive enzyme (HpaII) and insensitive enzyme (MspI) and hybridized with wsu102. The star indicates the fragment showing differences in methylation patterns. The genomic fragments identified by physical mapping are depicted as A, B and D on the left of the autoradiograph.
